# Cathepsins and cancer risk: a Mendelian randomization study

**DOI:** 10.3389/fendo.2024.1428433

**Published:** 2024-05-31

**Authors:** Tingting Deng, Xixue Lu, Xuemin Jia, Jinxin Du, Lijuan Wang, Baorui Cao, Meina Yang, Ying Yin, Fanjie Liu

**Affiliations:** ^1^ College of Traditional Chinese Medicine, Shandong University of Traditional Chinese Medicine, Jinan, China; ^2^ Bone Biomechanics Engineering Laboratory of Shandong Province, Shandong Medicinal Biotechnology Center (School of Biomedical Sciences), Neck-Shoulder and Lumbocrural Pain Hospital of Shandong First Medical University, Shandong First Medical University & Shandong Academy of Medical Sciences, Jinan, China; ^3^ National Health Commission (NHC) Key Laboratory of Biotechnology Drugs (Shandong Academy of Medical Sciences), Biomedical Sciences College, Shandong First Medical University, Jinan, China; ^4^ Department of Endocrinology, The First Affiliated Hospital of Shandong First Medical University, Jinan, China; ^5^ Department of Acupuncture, Affiliated Hospital of Shandong University of Traditional Chinese Medicine, Jinan, China

**Keywords:** cathepsins, cancers, Mendelian randomization, causality, single nucleotide polymorphisms (SNPs)

## Abstract

**Background:**

Previous observational epidemiological studies reported an association between cathepsins and cancer, however, a causal relationship is uncertain. This study evaluated the causal relationship between cathepsins and cancer using Mendelian randomization (MR) analysis.

**Methods:**

We used publicly available genome-wide association study (GWAS) data for bidirectional MR analysis. Inverse variance weighting (IVW) was used as the primary MR method of MR analysis.

**Results:**

After correction for the False Discovery Rate (FDR), two cathepsins were found to be significantly associated with cancer risk: cathepsin H (CTSH) levels increased the risk of lung cancer (OR = 1.070, 95% CI = 1.027–1.114, *P* = 0.001, *P_FDR_* = 0.009), and CTSH levels decreased the risk of basal cell carcinoma (OR = 0.947, 95% CI = 0.919–0.975, *P* = 0.0002, P*_FDR_* = 0.002). In addition, there was no statistically significant effect of the 20 cancers on the nine cathepsins. Some unadjusted low P-value phenotypes are worth mentioning, including a positive correlation between cathepsin O (CTSO) and breast cancer (OR = 1.012, 95% CI = 1.001–1.025, *P* = 0.041), cathepsin S (CTSS) and pharyngeal cancer (OR = 1.017, 95% CI = 1.001–1.034, *P* = 0.043), and CTSS and endometrial cancer (OR = 1.055, 95% CI = 1.012–1.101, *P* = 0.012); and there was a negative correlation between cathepsin Z and ovarian cancer (CTSZ) (OR = 0.970, 95% CI = 0.949–0.991, *P* = 0.006), CTSS and prostate cancer (OR = 0.947, 95% CI = 0.902–0.944, *P* = 0.028), and cathepsin E (CTSE) and pancreatic cancer (OR = 0.963, 95% CI = 0.938–0.990, *P* = 0.006).

**Conclusion:**

Our MR analyses showed a causal relationship between cathepsins and cancers and may help provide new insights for further mechanistic and clinical studies of cathepsin-mediated cancer.

## 1 Introduction

Cathepsins are a class of proteases found in various animal tissues intracellular (particularly in the lysosomal fraction). They finely regulate biological processes, such as proteolysis, metabolite storage, foreign body removal, immune response, and apoptosis, through efficient, highly selective, and limited specific substrate cleavage, thereby maintaining normal body homeostasis. However, irregularities in protein hydrolysis activity or “imbalances” of insufficient protease activity or excessive protein hydrolysis or dysregulation of signaling pathways are causative factors in diseases (1), including cancer, cardiovascular diseases, inflammatory and autoimmune diseases (2). A variety of catalytically active cathepsins act as potent effectors that alter the tumour microenvironment by remodeling the extracellular matrix (ECM) (at neutral pH), as well as the activation, processing, or degradation of chemokines, cytokines, and growth factors (3, 4). They also promote tissue invasion and metastasis by releasing cell adhesion molecules (5, 6) and are part of a dynamic response to anticancer therapy in the tumour microenvironment (7–9).

Recent studies have revealed the role of several cathepsins in promoting or inhibiting various cancers (e.g., lung (10), ovarian (11), thyroid (12), and colorectal (13)), including cathepsin B (CTSB) (14), cathepsin L (CTSL) (15), cathepsin G (CTSG) (16), and cathepsin S (CTSS) (17). However, few observational studies and clinical trials have investigated the relationship between cathepsins and cancer. Previous studies reported the high CTSB expression in pancreatic ductal adenocarcinoma (PDAC) cells in serum samples from patients with PDAC (18). One study found that the serum cystatin/CTSB ratio was a prognostic indicator of survival in patients with esophageal cancer (19). CTSS levels are significantly elevated in the sera of patients with gastric, esophageal, liver, colorectal, nasopharyngeal, and lung cancers (20). Despite extensive research, no uniform or conclusive study has been conducted on the correlation between cathepsins and cancer. Therefore, there is a need for further research on the causal relationship between the different types of cathepsins and cancer risk.

Mendelian randomization (MR) uses exposure-related genetic variants as instrumental variables (IVs) to robustly assess causality between exposure and outcome (21, 22). As alleles are randomly assigned and do not change in response to disease onset, MR analyses effectively reduce the influence of confounding factors, avoid reverse causation bias, and yield more reliable causal effects than observational studies (23, 24). MR analysis is now widely used to explore causal associations between exposure factors and cancer (25, 26). In oncology, MR analysis can provide insight into the complex relationship between exposure factors and cancer development, providing a basis for prevention and treatment in clinical research (27). Therefore, this study collected data on nine cathepsins and cancers from a large-scale genome-wide association study (GWAS), performed two-sample MR, followed by inverse MR to adjust for the pleiotropic effects of genetic tools and potential confounders, and assessed potential genetic-causal associations between cathepsins and cancers to provide a basis for future prevention and treatment strategies.

## 2 Materials and methods

### 2.1 Study design

A GWAS was performed for nine cathepsins and 20 cancers from the IEU GWAS database (https://gwas.mrcieu.uk/) at the University of Bristol, UK. Cathepsin data were obtained from an INTERVAL study, which included 3,301 Europeans (28). All donors completed a trial consent form, and the INTERVAL study was approved by the US National Research Ethics Committee (11/EE/0538). Considering the effect of linkage disequilibrium (LD) among SNPs, we screened for SNPs that were independent of each other and had genome-wide significance in the strength of association with cathepsin from the pooled GWAS data of cathepsin using the following screening criteria (29): (1)*P* < 5×10^–6^ of the correlation effect between cathepsin and IVs; (2) the physical distance between every two genes > 10,000 kb; and (3) R^2^ < 0.001 for LD between genes.

### 2.2 Data source

The GWAS summary statistics for a wide range of cancers were obtained from publicly available databases from the MRC IEU OpenGWAS (MR-base) database. We identified 20 cancer outcomes: bladder, lung, anal, testicular, thyroid, colorectal, ovarian, prostate, breast, esophageal, pharyngeal, endometrial, pancreatic, cecum, sialadenitis, hepatocellular, vulvar, gastric, basal cell, and bronchogenic carcinomas. The number of cases ranged from 105 to 122,188 (**Supplementary Table S1**).

### 2.3 Selection of IVs

**Figure 1:**
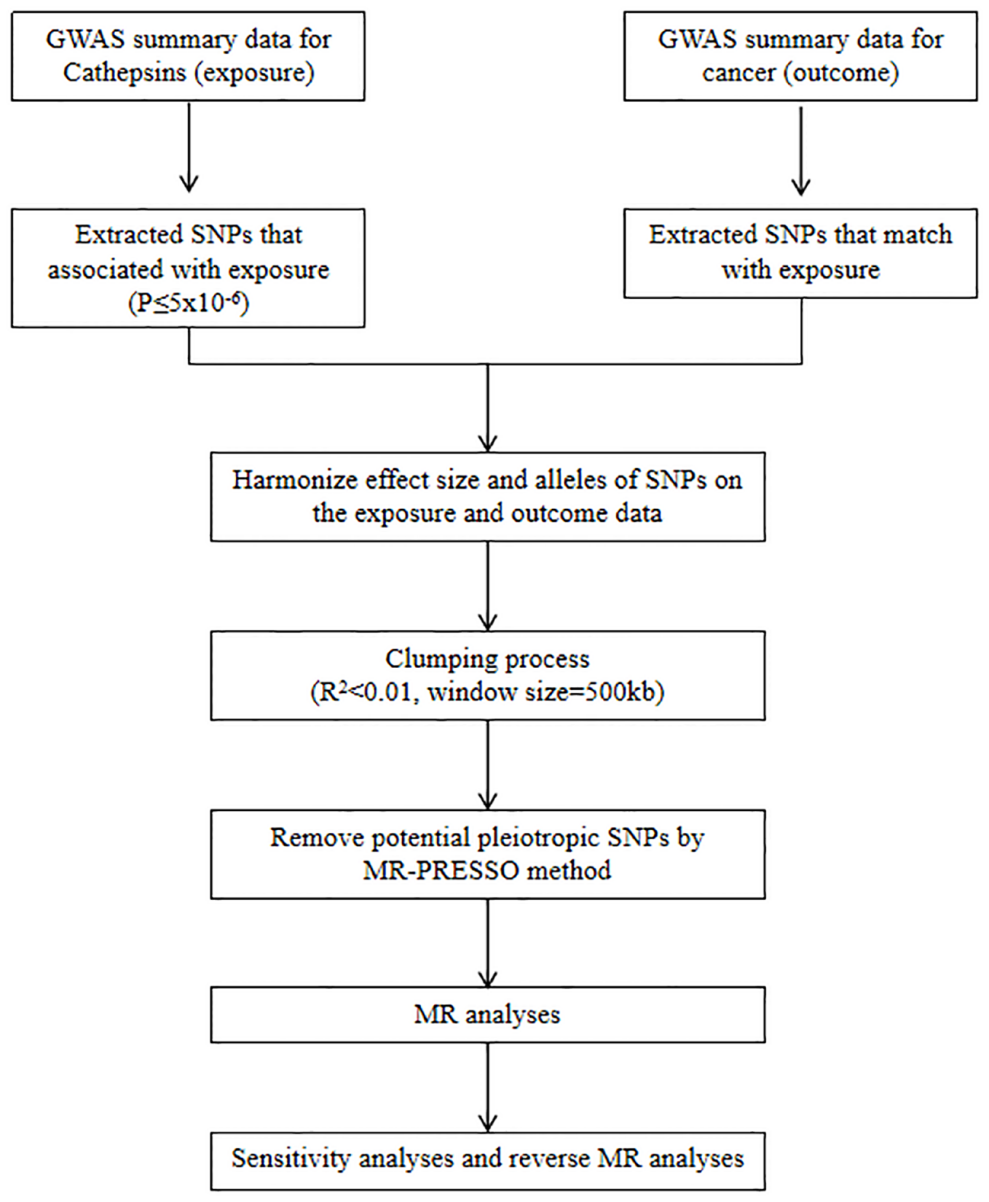
Study design and workflow.

We refer to the three core assumptions of association, independence, and exclusivity, which must be fulfilled in MR analyses. Single nucleotide polymorphisms (SNPs) with the genome-wide significance of association strength with cathepsins were selected as IVs. Weak IV bias was determined using the F-test statistic, and no weak IV bias was considered to exist if F > 10. The F statistic was calculated as F = [(N-K-1)/K]×[R^2^/(1-R^2^)], where N is the sample size, K is the number of IVs, and R^2^ denotes the variance of the exposure explained by each IV alone (30). A flowchart of the study is shown in **Figure 1**.

### 2.4 Statistical analysis

Determination of the causal relationship between cathepsins and cancer risk was carried out in two-sample Mendelian randomization using five methods: Inverse variance weighting (IVW) (31), MR-Egger (32), Weighted Median (33), Simple Mode (34), and Weighted Mode (35). Odds ratios (OR) and 95% confidence intervals (CI) were used to determine whether a causal relationship existed between cathepsins and cancer risk. According to previous studies, the IVW method is superior to other tests (36, 37), and is used as the main MR analysis method (38–40). Among these, the IVW was used as the primary method of analysis. Because of the multiple exposures and outcomes in this study, multiple test corrections were performed using the FDR method (41), and it was necessary to report whether the *P*-values tested by the IVW method reached nominal significance (*P* < 0.05) and statistical significance (*P_FDR_* < 0.05). The MR-Egger intercept was used to assess the relationship between IVs and other potential confounders and to ensure that the selected IVs did not influence the outcome variables through pathways other than exposure factors. Horizontal pleiotropy (27) is indicated if the MR-Egger intercept analysis shows a statistically significant relationship (*P* < 0.05). At *P* < 0.05, an outlier test was used to eliminate horizontal pleiotropy using the MR-PRESSO global test (42). An OR less than 1 indicates that exposure plays a protective role in predicting the occurrence of an outcome event. In other words, exposure played a positive role in preventing or reducing the occurrence of outcome events. Conversely, if the OR is greater than 1, the exposure is categorized as a risk factor for the outcome, and exposure can promote the occurrence of the outcome. Cochran’s Q statistic was used to perform the heterogeneity test. Statistically significant (*P* < 0.05) Cochran’s Q test proves that the analyses were significantly heterogeneous (43).

We performed a reverse MR analysis (20 cancers as exposures and cathepsins as outcomes) to explore whether cancer has a causal effect on cathepsins identified in the forward MR analysis. The analysis procedure was consistent with that of the forward MR analysis.

MR analyses were performed using “TwoSampleMR” (version 0.5.6) in R (version 4.2.3), Mendelian Randomization (0.7.0), and TwoSample MR (0.5.6). *P* < 0.05 indicates that the results are statistically significant.

## 3 Results

### 3.1 IVs selection

Based on the screening criteria, nine IVs for cathepsin were included in this study. The F-statistic for each IV was > 10, indicating low evidence of weak IV bias (**Supplementary Data 1**).

### 3.2 MR main analysis results

**Figure 2:**
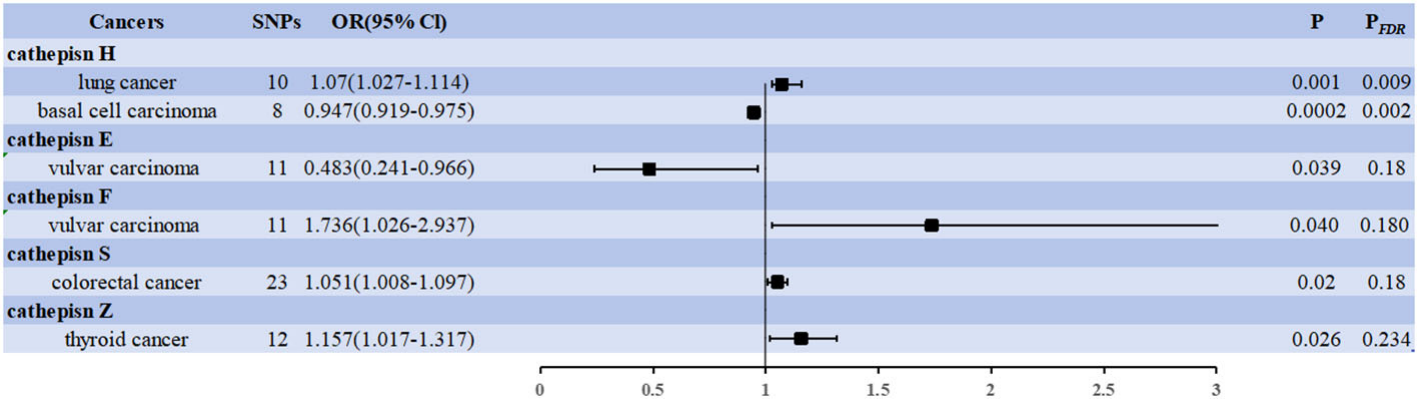
Forest plots showed the causal associations between cathepsins and cancers. IVW, inverse variance weighting; CI, confidence interval; FDR, false discovery rate.

The IVW approach revealed significant evidence of a causal relationship between cathepsins and different cancer risks. Our pooled analysis identified nine cathepsins that exhibited potential causal associations with 20 cancers (**Figure 2**). Of the 180 associations included (9 exposures x 20 outcomes), six were statistically significant in the IVW analysis (**Figure 2**). Cathepsin H (CTSE) levels reduced the risk of vulvar carcinoma (OR = 0.483, 95% CI = 0.241–0.966, *P* = 0.039), and cathepsin H (CTSH) levels reduced basal cell carcinoma risk (OR = 0.947, 95% CI = 0.919–0.975, *P* = 0.0002); CTSF levels increased the risk of vulvar carcinoma (OR = 1.736, 95% CI = 1.026–2.937, *P* = 0.040), CTSS levels increased the risk of colorectal cancer (OR = 1.051, 95% CI = 1.008–1.097, *P* = 0.02), CTSZ levels increased the risk of thyroid cancer (OR = 1.157, 95% CI = 1.017–1.317, *P* = 0.026), CTSH levels increased the risk of lung cancer (OR = 1.070, 95% CI = 1.027–1.114, *P* = 0.001).

Two associations were based on the number of exposure-outcome pairs showing FDR-corrected significance (*P* < 0.05). CTSH levels increased the risk of lung cancer (OR = 1.070, 95% CI = 1.027–1.114, *P* = 0.001, *P_FDR_* = 0.009), and CTSH levels decreased the risk of basal cell carcinoma (OR = 0.947, 95% CI = 0.919–0.975, *P* = 0.0002, *P_FDR_* = 0.002). These two associations had 10 and 8 IVs, respectively, and the robustness of these causal relationships was further supported by combined data from multiple sensitivity analyses (**Supplementary Data 2**). Specifically, our analyses by Cochran’s Q did not reveal any signs of heterogeneity (*P* = 0.729 > 0.05, *P* = 0.065 > 0.05). The MR-Egger intercept assessment did not provide evidence of horizontal pleiotropy (*P* = 0.236 > 0.05, *P* = 0.969 > 0.05).

### 3.3 Reverse MR analysis results

**Figure 3:**
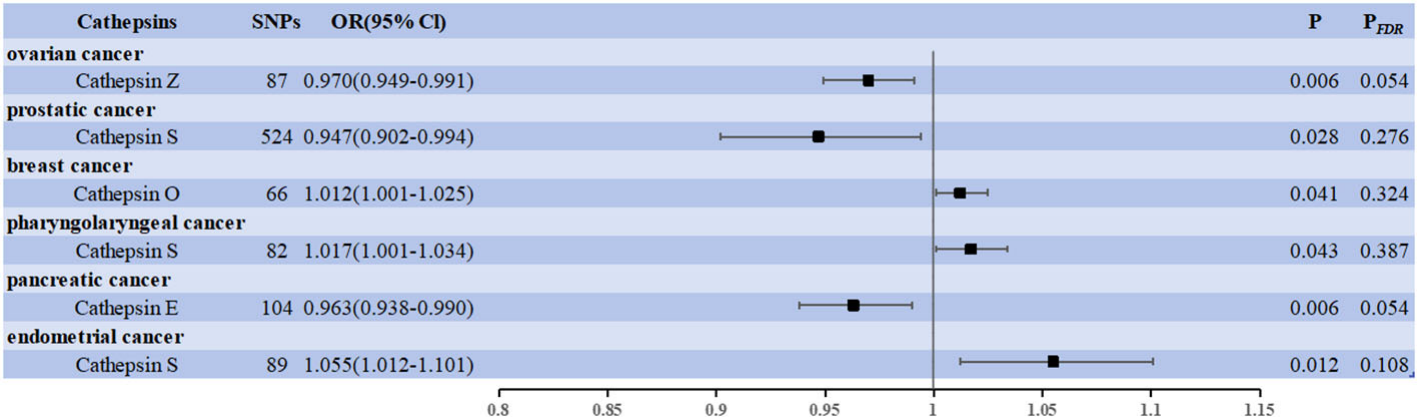
Forest plots showed the causal associations between cancers and cathepsins. IVW, inverse variance weighting; CI, confidence interval; FDR, false discovery rate.

We used cancer as the exposure factor, cathepsins as the outcome, and cancer-associated SNPs (*P* < 5 × 10–^5^) as the IVs to explore whether there was reverse causality for the significant results obtained. **Figure 3** shows the six cathepsin immunophenotypes potentially affected by cancer. After reverse analysis, six were statistically significant: a positive correlation between CTSO and breast cancer (OR = 1.012, 95% CI = 1.001–1.025, *P* = 0.041), CTSS (OR = 1.017, 95% CI = 1.001–1.034, *P* = 0.043) and pharyngeal cancer, and CTSS (OR = 1.055, 95% CI = 1.012–1.101, *P* = 0.012) and endometrial cancer; There was a negative correlation between CTSZ and ovarian cancer (OR = 0.970, 95% CI = 0.949–0.991, *P* = 0.006), CTSS and prostate cancer (OR = 0.947, 95% CI = 0.902–0.944, *P* = 0.028), CTSE and pancreatic cancer (OR = 0.963, 95% CI = 0.938–0.990, *P* = 0.006). The Cochrane Q-test provided no evidence of heterogeneity (*P* = 0.388 > 0.05, *P* = 0.837 > 0.05; *P* = 0.909 > 0.05, *P* = 0.221 > 0.05, *P* = 0.667 > 0.05, *P* = 0.667 > 0.05, *P* = 0.832 > 0.05). SNP pleiotropy was not detected for the MR-Egger test intercept (*P* = 0.872 > 0.05, *P* = 0.393 > 0.05, *P* = 0.695 > 0.05; *P* = 0.200 > 0.05, *P* = 0.558 > 0.05, *P* = 0.290 > 0.05). These associations, based on the number of exposure-outcome pairs, did not show FDR-corrected significance (*P* > 0.05) (**Figure 3**). The results of the heterogeneity and pleiotropy tests are presented in **Supplementary Data 3**.

## 4 Discussion

This study investigated the causal association between cathepsin levels and cancer. The causal effects of nine cathepsins on 20 cancers were comprehensively evaluated by MR analysis. The results showed a causal association between certain cathepsins and cancers, suggesting that cathepsins may have an essential influence on cancer and play an important role in cancer development.

In recent decades, the incidence of various types of cancer has increased; cancer has become a significant public health problem worldwide. It is the second leading cause of death in humans, after cardiovascular diseases (44). CTSH acts as an aminopeptidase and endopeptidase with endo protein hydrolytic activity and can hydrolyze a wide range of proteins (45). CTSH has been detected in type II pneumocytes and alveolar macrophages in the lung (46, 47). It is located in lamellipodia, dense multivesicular vesicles, and type II complex vesicle pneumocytes, which constitute sites of surfactant maturation (48, 49). Microarray analysis studies have shown that CTSH expression is lower in non-small cell lung cancer than in normal lung tissue (50) and that CTSH is involved in SP-B maturation by cleaving the peptide bond between pro-SP-B residues 279 and 280 (51, 52). Some studies have also found that silencing of CTSH significantly reduces SP-B maturation and subsequently reduces SP-B secretion (53). CTSH progression in lung cancer may regulate the sPLA2-PKCδ-MAPKs-cPLA2α pathway by modulating SP-B maturation, thereby regulating lipid metabolism in the lungs (54, 55). CTSH is highly expressed in small cells and in adenocarcinomas (56, 57). Luyapan et al. (58) conducted a transcriptome-wide association study using expression weights from a quantitative trait locus study of lung expression and found that the gene most strongly associated with lung cancer was CTSH.

The epidermis of the skin constantly undergoes cell renewal and differentiation to maintain its normal structure and function. However, when the balance between renewal and differentiation is disrupted, uncontrolled cell proliferation and cancer can result (59). Basal cell carcinoma, the most common form of skin cancer, originates in the basal layer of the epidermis and appendages. The tumor grows slowly, rarely metastasizes, and generally infiltrates the surrounding tissues slowly (60). The interplay between various environmental, genetic, phenotypic, and genetic risk factors contributes to the development of basal cell carcinomas. Cathepsin is an essential protease required for invasion. It has been found that CTSH is mainly localized in the lowermost basal cell layer (61). Basal cells are undifferentiated and can grow and divide. CTSH is a lysosomal cysteine protease involved in the degradation of extracellular matrix components and has been found to be more active in basal cell carcinoma tumors than in normal skin tissue (62). The mechanism underlying the involvement of CTSH in the development of basal cell carcinoma has not yet been investigated. However, CTSH activity is dysregulated in tissues surrounding basal cell carcinoma tumors, leading to its overexpression and secretion into the extracellular space to degrade structural proteins such as collagen and fibronectin (8, 63–67), thereby regulating the structure and stability of the extracellular matrix and promoting tumor cell invasion (68, 69).

It is also worth noting that breast cancer was associated with elevated CTSO, pharyngeal and endometrial cancers with elevated CTSS, ovarian cancer with decreased CTSZ, prostate cancer with decreased CTSS, and pancreatic cancer with decreased CTSE. CTSO was found to be significantly overexpressed in T47D, CAMA-1, and ZR75–1 cells, reducing BRCA1 levels and promoting cell proliferation by promoting the cysteine protease-mediated degradation of metadherin, polyadenylate-binding protein 4-like, recombinant lamin A/C, and recombinant eukaryotic translation elongation factor 1 alpha 1 protein levels (70–72). However, CTSS and CTSE are overexpressed in prostate cancer (73) and pancreatic cancer (74), respectively; this is contrary to the results of the present study and needs to be verified by more clinical and experimental studies in the future.

Previous studies did not comprehensively analyze the causal relationship between cathepsins and cancer. This study used two samples of MR studies and obtained reliable results: firstly, MR analysis has the advantage of avoiding reverse causal associations and confounders and saving time and resources compared to observational studies; secondly, according to our analysis, multiple cathepsins are risk and protective factors for cancers, and this study did not reveal potential horizontal pleiotropy, thus confirming the reliability of the conclusions. However, there are some limitations to this study. First, this study only observed a causal effect of cathepsins on the risk of multiple cancers at the gene level. Future MR studies with larger sample sizes and randomized controlled trials are required to validate these results. Second, the study was limited to the European population, and it is not possible to demonstrate whether the findings can be extended to other populations. Furthermore, as with all published MR studies, the possibility that unobserved pleiotropy affects the results cannot be ruled out, even if measures are taken to identify and eliminate aberrant variants (21); the study was unable to infer a non-linear correlation between cathepsins and cancers. Lastly, the cathepsins and cancer GWAS data were obtained from publicly available databases, and subgroup analyses were not possible due to the lack of detailed clinical patient information. In summary, the results of this study, using two-sample and inverse MR methods, suggest a causal relationship between cathepsins and various cancers. The results of this study should be interpreted with caution. More investigative studies should be conducted to validate the results and consider their application in clinical trials.

## 5 Conclusion

In conclusion, these results suggest a potential causal relationship between cathepsins and cancer. These findings provide new insights for further mechanistic studies on cathepsin-mediated cancers, potential targets, and new biomarkers for the early diagnosis and interventional therapy of cancers.

## Data availability statement

The original contributions presented in the study are included in the article/**Supplementary Material**. Further inquiries can be directed to the corresponding authors.

## Ethics statement

Ethical approval was not required for the study involving humans in accordance with the local legislation and institutional requirements. Written informed consent to participate in this study was not required from the participants or the participants’ legal guardians/next of kin in accordance with the national legislation and the institutional requirements.

## Author contributions

TD: Conceptualization, Methodology, Writing – original draft. XL: Visualization, Writing – original draft. XJ: Data curation, Project administration, Writing – original draft. JD: Data curation, Project administration, Writing – original draft. LW: Software, Writing – original draft. BC: Formal Analysis, Investigation, Writing – original draft. MY: Funding acquisition, Writing – review & editing. YY: Resources, Supervision, Writing – review & editing. FL: Writing – review & editing.
